# Impact of kidney transplantation on functional status

**DOI:** 10.1080/07853890.2021.1962963

**Published:** 2021-08-13

**Authors:** Hatem Ali, Karim Soliman, Mahmoud M. Mohamed, Manzur Rahman, Johann Herberth, Tibor Fülöp, Ingi Elsayed

**Affiliations:** a Renal Department, University Hospitals of Coventry and Warwickshire, Coventry, UK; b Department of Medicine, Division of Nephrology, Medical University of South Carolina, Charleston, SC, USA; c Department of Medicine, Division of Nephrology, Cairo University, Giza, Egypt; d Department of Medicine, Division of Nephrology, University of Tennessee Health Science Center, Memphis, TN, USA; e Department of Renal Medicine, Royal Stoke University Hospitals, Stoke-on-Trent, UK; f Medicine Services, Ralph H. Johnson VA Medical Center, Charleston, SC, USA

**Keywords:** Frailt, steroids, outcome

## Abstract

**Background and aims:**

Functional capacity (FC) is known to affect morbidity and mortality in kidney transplantation. Despite this important role, little is known about the variables influencing post-transplant FC. Our study aims at identifying these crucial associations.

**Method:**

Our study included 16,684 renal transplant recipients (RTR). Patients had transplant between 1 September 2018 and 1 September 2019. Mild functional impairment was defined as those with a KPSS score > or = 80; moderate functional impairment was defined as those with a KPSS score between 50 and 70 and severe functional impairment was defined as those with a KPSS score < or =40. The outcome measured was FC at follow-up one-year post-transplant. Abnormal FC at follow-up was defined as those with KPSS score less than 80%. Normal FC at follow-up was defined as those with KPSS score equal or above 80%. Multivariate logistic regression was used to assess with the relationship between patient characteristics and abnormal functional status post-transplant.

**Results:**

Three groups were identified; those with none-to-mild functional impairment at time of transplant (Group A; *n* = 8388), those who had moderate impairment at time of transplant (Group B; *n* = 7694) and those who had severe impairment at time of transplant (Group C; *n* = 602). Abnormal FC at one-year post transplant was present in 7.69%, 28.89%, 49.49% of patients in group A, B and C, respectively. Glucocorticoid withdrawal was associated with lower risk of developing abnormal FC post-transplant (OR = 0.75, *p* value = .02, 95% confidence intervals: 0.64 to 0.97), while recipient diabetes was associated with higher risk of abnormal FC (OR = 1.44, *p* value <.01, 95% confidence intervals: 1.20 to 1.74) in adjusted model.

**Conclusion:**

Kidney transplantation is associated with substantial improvement in all stages of FC in KTRs. Glucocorticoid withdrawal and diabetes mellitus are potentially modifiable factors of FC and merit further considerations during pre-transplant workup and post-transplant immunosuppressive therapeutic planning.

**Key messages**
- Kidney transplantation is associated with substantial improvement in all stages of FC in KTRs.
- Glucocorticoid withdrawal and diabetes mellitus are potentially modifiable factors of FC and merit further considerations during pre-transplant workup and post-transplant immunosuppressive therapeutic planning.

## Introduction

Frailty is defined as a state of decreased physiologic reserve and diminished ability to recover from physiologic stressors [1]. Functional capacity (FC) is a major component of frailty, a pivotal determinant of health and thought to be a reliable indicator of quality of life [2]. Many transplant centres have incorporated FC level assessments to determine candidacy for transplant by using tools such as the Karnofsky Performance Status Scale (KPSS) or the Sickness Impact Profile [3]. During and after medical illness or intervention, FC may decline significantly. Failure to recover places individuals at higher risk for complications reaching up to mortality [4].

Kidney transplantation (KT) is the best treatment option with superior survival for patients with end-stage kidney disease (ESKD). However, data are limited regarding potential influencing factors on FC and its outcome in kidney transplant recipients (KTR) [5]. Chronic immunosuppression administration, particularly glucocorticoid and calcineurin inhibitors (CNIs), has deleterious effects on muscle metabolism, bone mass, development or worsening of diabetes and increased risk of infections, including sepsis. All of these factors are expected to negatively affect FC [6,7].

Studies demonstrate that frailty is associated with a two-fold increased risk of delayed graft function, 1–2-fold increased risk of protracted initial hospital stay and 1–6-fold increased risk of hospital readmission within one month post-transplant [8]. Additionally, pre-transplant hospitalization, a surrogate measure of frailty, is also associated with increase post-transplant hospitalizations [9]. Moreover, ESKD impacts negatively FC by the detrimental effects of uraemic syndrome, lack of energy and loss of time on dialysis, limiting availability to exercise or work [5]. A comprehensive understanding of FC in pre- and post-RT is crucial for clinicians and potential KTRs who often pursue transplantation with a principal hope to restore functional status and quality of life [5]. Thus, the aim of this study is to examine the effect of KT-associated variables on post-transplant FC.

## Methodology

### Data extraction

Using data from the United States Organ Procurement and Transplantation Network (OPTN) from 1 September 2018 till 1 September 2019, we retrospectively reviewed all renal transplant patients who had FC assessments at the time of transplant and one-year post-transplant. Data including age, gender, ethnicity, functional status, diabetes (diagnosed before the date of transplantation), body mass index, cold ischaemia time, number of previous transplants, panel reactive antibodies, donor type, donor age, HLA-mismatches, number of acute rejection episodes, induction therapies, maintenance immunotherapy on discharge were collected. The outcome measured was FC one-year post-transplant and was classified according to KPSS score. Exclusion criteria were: patients less than 18 years old, organ transplantations other than the kidneys, multiple organ transplantations, patients who were discharged on non-tacrolimus-based immunotherapy, patients with missing FC assessment at time of transplant or one-year post-transplant and patients who had FC assessments by methods other than KPSS. The study was exempted from institutional approval as it was performed with publicly available, de-identified data.

### Study definitions and outcomes

KPSS measures the patient’s ability to perform ordinary tasks on a scale of 0 to 100. FC measurement definitions are outlined in Appendix Table 1 in the Supplementary data. None-to-mild functional impairment was defined as those with a KPSS score ≥80. Moderate functional impairment was defined as those with a KPSS score between 50 and 70. Severe functional impairment was defined as those with a KPSS score KPSS score ≤40. The outcome measured was FC at follow-up one-year post-transplant. The main hypothesis of our research was that among renal transplant patients, the majority will have normal functional status (which we defined as a KPSS score ≥ 80). Contrarily, abnormal FC at follow-up was defined as those with KPSS score less than 80. In this paper, nomenclature to describe kidney-associated variables is used in keeping with last KDIGO consensus statement [10].

**Table 1.** Baseline characteristics for the groups included in the study.

	None to mild functional impairment (*n* = 8388)	Moderate functional impairment (*n* = 7694)	Severe functional impairment (*n* = 602)	*p* Value
Recipient age:	52.01 (14.00)	54.69 (13.16)	54.96 (12.24)	<.01
Mean (SD)				
Sex:Male (N,%)	5197 (61.95%)	4663 (60.60%)	376 (62.45%)	.18
Ethnicity (N,%)				<.01
White	4137 (49.32%)	3117 (40.51%)	307 (50.99%)	
Black	1904 (22.69%)	2527 (32.84%)	146 (24.25%)	
Hispanic	1529 (27.06%)	1319 (17.14%)	105 (17.44%)	
Asian	609 (7.26%)	551 (7.16%)	31 (5.14%)	
American Indian	73 (0.87%)	61 (0.79%)	7 (1.16%)	
Native Hawaiian	39 (0.46%)	36 (0.46%)	3 (0.49%)	
Multiracial	97 (1.15%)	83 (1.07%)	3 (0.49%)	
Recipient BMI (kg/m^2^):	28.71 (5.62)	29.02 (5.74)	28.89 (8.60)	<.01
Mean (SD)				
Dialysis before transplant: Yes (N,%)	2643 (31.50%)	2512 (32.64%)	174 (28.90%)	<.01
HLA mismatch:	4.02 (1.52)	4.15 (1.42)	4.43 (1.23)	<.01
Mean (SD)				
Extended criteria donor: Yes (N,%)	772 (9.20%)	887 (11.52%)	72 (11.96%)	.06
Glucocorticoid withdrawal protocol: Yes (N,%)	2853 (34.01%)	2043 (26.55%)	102 (16.94%)	<.01
Serum creatinine at discharge (mg/dL): Median (25% and 75% IQR)	2.09 (1.3, 4.6)	2.53 (1.44, 5.5)	1.6 (0.97, 3.31)	<.01

### Statistical analysis

After removing duplicates from the STARFILES, the files were merged using 1:m mode. Categorical variables were described in numbers and percentages. Continuous variables were described as means and standard deviation. Chi-square analysis was used for comparison between categorical variables, while Kruskal–Wallis test was used for comparison between continuous variables. T independent test was used to compare means of KPSS score at the time of transplant and at one-year post-transplant. Multivariate logistic regression analysis was used to assess the relationship between patient characteristics and abnormal functional status post-transplant. Variables that had a *p* value less than or equal to.05 in the univariate logistic regression analysis were included in the multivariate model. Backward stepwise approach was used to get the best fit for the multivariate logistic regression model. In order to perform the backward stepwise approach, variables with *p* value more than .2 were removed from the model. Scaled Schoenfeld residuals were used to assess for proportionality assumption for each variable and for the whole model. Variables assessed in the univariate analysis were: recipient age, recipient sex, recipient ethnicity, diabetes, time on maintenance dialysis pre-transplant, recipient body mass index (BMI), donor type (extended criteria donor or not), corticosteroid withdrawal post-transplant and post-operative serum creatinine at the time of discharge. These variables were chosen based on clinical judgement. Hosmer–Lemeshow goodness-of-fit analysis was performed to assess calibration of the models. Sensitivity of the model was assessed using area under the curve (AUC) analysis. Linktest was used to assess the specification error. A *p* value less than or equal to .05 was the cut-off point for identifying poor fit or specification error for the model. To perform a sensitivity analysis, we performed a subgroup analysis among elderly population. As elderly patients aged 65 years or above are more prone to have abnormal functional performance, we performed a subgroup analysis using the same multivariate logistic regression analysis (with the same variables) on this subgroup of patients.

## Results

**Figure 1.**
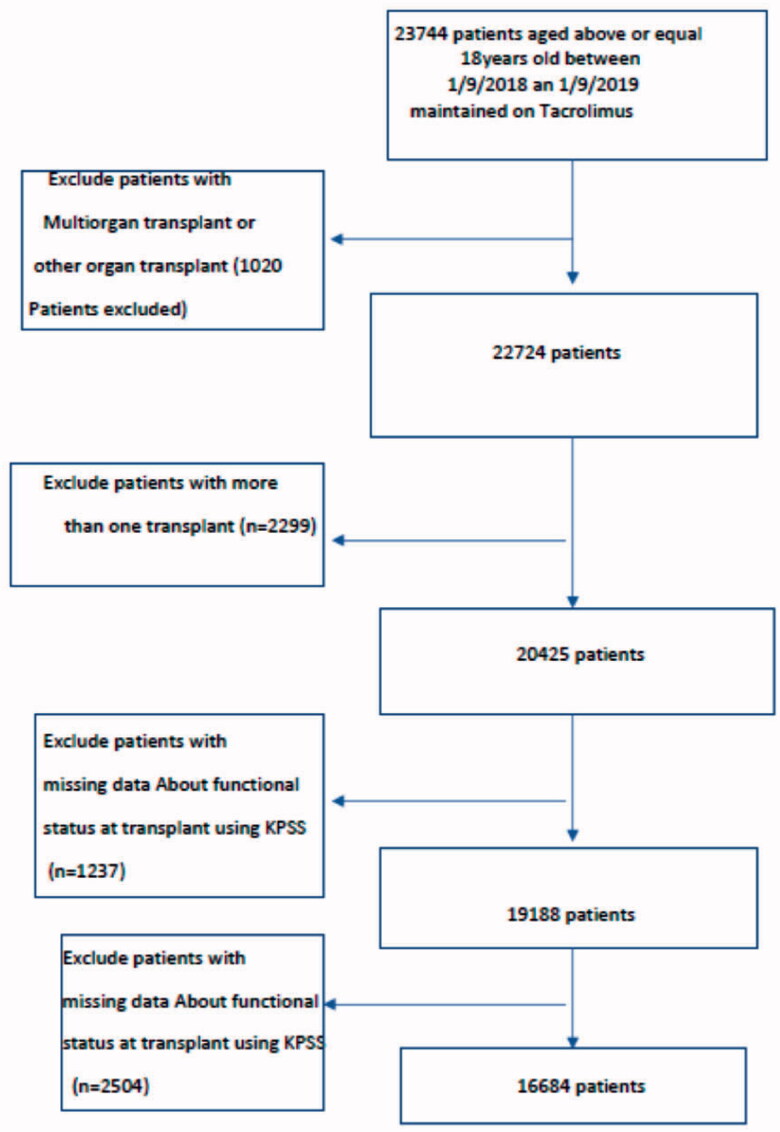
Hierarchy for selection of patients from the STARFILES.

A total of 16,684 patients were included in our study. Details of selecting patients from the cohort are shown in Figure 1. Three groups were identified; those who had ≤ mild functional impairment at the time of transplant (Group A; *n* = 8388), those who had moderate impairment at the time of transplant (Group B; *n* = 7694) and those who had severe impairment at the time of transplant (Group C; *n* = 602). Baseline characteristics of the identified groups are shown in Table 1.

There was significant improvement in KPSS score at one-year post transplant (mean = 84.41%, confidence interval: 0.842 to 0.846) in comparison to KPSS score at the time of transplant (mean = 73.68%, confidence intervals: 0.734 to 0.738) with *p* value < .001. Out of 16,684 patients included in our study, 13,516 patients had normal FC at one-year post transplant (81.01%), while only 3168 patients had abnormal FC through the same time period (18.99%). Abnormal FC at one-year post transplant was present in 7.69%, 28.89%, 49.49% of patients in group A, B and C, respectively. Details of changes in functional status at transplant and at follow-up are shown in Table 2.

**Table 2.** Details of changes in functional status at the time of transplant and at follow-up.

	None to mild functional impairment at follow-up	Moderate functional impairment at follow-up	Severe functional impairment at follow-up
None to mild functional impairment at transplant	7742 (92.99%)	555 (6.61%)	91 (1.08%)
(*n* = 8388)			
Moderate functional impairment at transplant	5470 (71.09%)	2041 (26.52%)	183 (2.37%)
(*n* = 7694)			
Severe functional impairment at transplant	304 (50.49%)	194 (32.22%)	104 (17.27%)
(*n* = 602)			

Multivariate logistic regression analysis to assess variables associated with abnormal FC is shown in Table 3. AUC for this model was 0.61. There was no evidence of poor fit in this model (*p* = .27) as well as no evidence of specification error (*p* = .54) . Glucocorticoid withdrawal protocol (OR = 0.77, *p* value <.001, 95% confidence intervals: 0.64 to 0.80) and Asian ethnicity (OR = 0.71, *p* value < .001, 95% confidence intervals: 0.57 to 0.87) were associated with lower risk of developing abnormal FC. Extended criteria donors, time on dialysis (months), black ethnicity, recipient age and recipient diabetes were associated with increased risk of abnormal functional status at follow-up (OR = 1.14, 1.000044, 1.26, 1.01 and 1.45, respectively).

**Table 3.** Multivariate logistic regression analysis to assess variables associated with abnormal FC at one year from kidney transplant.

	Odds ratio	Standard error	*p* Value	95% Confidence interval
Glucocorticoid withdrawal	0.71	0.03	.00	0.64 to 0.80
Extended criteria donor	1.14	0.07	.04	1.002 to 1.29
Time on dialysis (months)	1.00	0.00	.04	1.000003 to 1.000085
Diabetes mellitus	1.45	0.07	.00	1.31 to 1.60
BMI at time of transplant (per unit change, kg/m^2^)	1	0.00	.13	0.99 to 1.01
Ethnicity:				
Black	1.26	0.07	.00	1.13 to 1.42
Hispanic	0.87	0.06	.07	0.75 to 1.01
Asian	0.71	0.07	.00	0.57 to 0.87
America Indian	0.98	0.23	.94	0.61 to 1.58
Pacific Islander	0.49	0.20	.08	0.22 to 1.09
Multi-racial	0.60	0.16	.06	0.35 to 1.03
Age (years)	1.01	0.00	.00	1.01 to 1.02

**Table 4.** Multivariate logistic regression analysis among patients equal to or above 65 years old to assess variables associated with abnormal FC at one year from kidney transplant.

	Odds ratio	Standard error	*p* Value	95% Confidence interval
Glucocorticoid withdrawal	0.79	0.08	.02	0.64 to 0.97
Extended criteria donor	0.93	0.09	.50	0.75 to 1.14
Time on dialysis (months)	1.00	0.00	.08	0.99 to 1.0001
Diabetes mellitus	1.44	0.13	.00	1.19 to 1.73
BMI at time of transplant (per unit change, kg/m^2^)	1.01	0.00	.04	1.0006 to 1.03
Ethnicity:				
Black	1.09	0.12	.41	0.87 to 1.36
Hispanic	0.97	0.13	.83	0.74 to 1.27
Asian	0.77	0.14	.17	0.53 to 1.11
America Indian	0.49	0.32	.27	0.14 to 1.75
Pacific Islander	1.64	1.21	.50	0.38 to 7.02
Multi-racial	0.41	0.32	.25	0.09 to 1. 87
Age (years)	1.02	0.01	.09	0.99 to 1.04

To perform a sensitivity analysis, we did the same multivariate logistic regression analysis among patients older than or equal to 65 years old. Results are shown in Table 4. Number of patients included in this model was 3966. Glucocorticoid withdrawal was associated with lower risk of developing abnormal FC post-transplant (OR = 0.79, *p* value = .02, 95% confidence intervals: 0.64 to 0.97). Diabetes mellitus was associated with higher risk of having abnormal FC post-transplant (OR = 1.44, *p* value <.01, 95% confidence intervals: 1.19 to 1.73). AUC for this model was 0.58. There was no evidence of poor fit with *p* value = .29 as well as no evidence of specification error with *p* value = .66.

## Discussion

Our retrospective study with a large number of patients from the OPTN database (*n* = 16,684) demonstrates that KT led to significant improvements in all stages of FC regardless of pre-transplant FC. Moreover, at one-year follow up and after performing sensitivity analysis, withdrawal of glucocorticoids was associated with significant improvements in FC, while baseline diabetes was associated with worsening FC. To our knowledge, this is the first study to show an effect of diabetes mellitus and glucocorticoid withdrawal on FC in KTRs maintained on tacrolimus therapy.

Reese et al. [11] utilized UNOS registry data to examine the effect of pre-transplant functional status (physical function scale of the Medical Outcomes Study Short Form-36) on KTR’s survival over a period of three years post-transplant. The authors concluded that functional status was an independent predictor of post-transplant survival [11]. Similarly, a study from Johns Hopkins and University of Michigan Hospitals utilizing physical and kidney-disease specific Health-Related Quality Of Life (HRQOL) scores post-KT (*n* = 443) showed improvement in both initially frail and non-frail recipients with more marked improvements noted in frail KTRs [12]. Our results support these findings and identify additional novel factors that influence FC outcome one-year post KT. Current guidelines do not specify a quantitative threshold for “frailty” at which a patient could be at higher risk for adverse post-transplant outcomes [13,14]. Recent Kidney Disease and Improving Global Outcomes (KDIGO) guidelines recommend evaluation of frailty at the time of listing and while being on the waitlist in order to determine transplant candidacy; however, no cut-off values have been proposed to date [15,16]. The results of our study could contribute to such a guideline discussion and form a base for future recommendations.

A major finding of our study is the positive association between glucocorticoid withdrawal and improved FC. Corticosteroid maintenance versus withdrawal protocols in KTRs remain subject of substantial controversy among transplant experts, with sifting trade-offs between risks and benefits of immunosuppressive therapy across the spectrum of baseline health [7]. Some favour its use arguing the long-term benefit of graft survival and lower incidence of rejection [17]. An opposing opinion states that chronic glucocorticoid use carries the risk of over-immunosuppression and potential side effects. These include increased rates of infections, hospitalizations, osteoporosis, cataracts, hypertension, development of diabetes post-transplant, all of which contribute to increased risk of mortality [18–25]. Historically, in the era of azathioprine/cyclosporine, the European Best Practice Guidelines for Renal Transplantation suggested that glucocorticoid withdrawal is safe only in low-risk patients provided that renal function is monitored carefully. Mirroring this opinion, KDIGO clinical practice guidelines suggested that glucocorticoids may be discontinued during the first week after transplantation in patients who are at low immunologic risk and who also received induction therapy [26]. An equivocal analysis from the United States Renal Data System showed that glucocorticoid-free maintenance immunosuppression was associated with a reduced risk of pneumonia, sepsis and diabetes but also with a higher risk of graft failure [27]. A large retrospective study of 4842 KTRs receiving CsA demonstrated no negative impact of steroid withdrawal on graft function or survival and was associated with a significant reduction in mortality [28]. In contrast, a Canadian steroid withdrawal study showed a significant benefit of glucocorticoid maintenance compared to withdrawal in regards to long-term graft survival and renal function but this study did not include patients on MMF/FK regimen [29–32]. Studies on MMF/FK-based immunosuppressive regimen showed lower incidence of acute rejection, enabling earlier and safer withdrawal of glucocorticoid administration [33]. A randomized study of glucocorticoid withdrawal three days after transplantation in patients treated with MMF, FK and induction therapy with daclizumab showed no significant differences in the incidence of acute rejection and renal function at one year compared with the control group [34]. In 2008, the results of the well-designed, long-term, prospective, randomized, controlled ASTELLAS study rekindled the steroid use debate favouring steroid maintenance therapy over withdrawal. The study included 386 KTRs divided into two groups (glucocorticoid maintenance versus withdrawal) [31]: Group 1 had prednisone stopped by 7 days post-transplant, while group 2 tapered to 5 mg per day by six months as maintenance therapy. At five years, there was no significant difference in patients reaching the composite end point of death, moderate/severe acute rejection, or graft loss. Potential glucocorticoid side effects were comparable in both groups in regards to blood pressure, new-onset diabetes, serum cholesterol, low-density lipoprotein levels and rates of bone fracture, however, with significant increase in triglyceride values and weight gain in group 2. Since ASTELLAS study did not examine FC, our study could complement the findings pertaining to steroid side effects described there assuming that weight gain and hypertriglyceridaemia, both negatively impact FC.

Several studies have proved adverse outcomes of diabetes and its impact on functional status of the patients. However, studies assessing the impact of diabetes on functional status post renal transplant are scarce. In our study, diabetes mellitus is one of the risk factors for abnormal FC post-transplant.

The mechanisms by which KT can improve post-transplant FC have not yet been defined. It was suggested that renal transplantation can play a role in improving heart failure and low ejection fraction [35]. We hypothesize that improvements in cardiac function could contribute to improvements in post-transplant FC. The results of our study suggest that functional status, and thus frailty, is not an irreversible status and can be improved after renal transplantation. Our study supports the results of previous studies performed by McAdams et al. [36] that showed the dynamic changes in the physiological reserve of patients’ post-renal transplant.

We want to acknowledge the limitations of our findings that include the retrospective study design, missing data about other comorbidities for renal transplant patients and the utilization of only one FC measurement tool (KPSS). Notwithstanding these, we present novel findings that may potentially have impact on the current management and selection strategies for RT: limiting corticosteroid use post-RT and adequate control of diabetes might contribute to improved post-transplant FC. Their contributions to this important clinical outcome in the MMF/FK era post-KT merit further study.

## Supplementary Material

Supplemental Material

## Data Availability

The data used in this study are publicly available in de-identified form in OPTN database.
