# Supplementary material for: Impact of kidney transplantation on functional status
Source: Ann Med. 2021 Aug 13;53(1):1303–9. doi: 10.1080/07853890.2021.1962963 (PMC8366639; doi:10.1080/07853890.2021.1962963)
Supplement: Supplemental Material [file IANN_A_1962963_SM2366.docx]

| **General category** | **Score** | **Karnofsky scale** |
| --- | --- | --- |
| -Able to carry on normal activity, no special care needed | 100 | Normal, no complaints or no evidence of disease |
|  | 90 | Able to perform normal activities, minor evidence of disease |
|  | 80 | Able to perform normal activities with effort, some evidence of disease |
| -Unable to work  -Able to live at home and care for most personal needs  -Various amounts oof assistance needed | 70 | Cares for self but unable to carry on normal activity or active work |
|  | 60 | Requires occasional assistance but able to care for needs |
|  | 50 | Requires considerable assistance and frequent medical care |
| -Unable to care for self  -Requires institutional or hospital care or equivilant  -Disease may be rapidly progressing | 40 | Disable, requires special care and assistance |
|  | 30 | Severely disabled, hospitalization is indicated |
|  | 20 | Very sick, hospitalization, active treatment necessary |
| -Terminal state | 10 | Fatal process accelerating rapidly |

**Table 1:** Karnofsky Performance Status scale measurements
